# Content Patterns in Topic-Based Overlapping Communities

**DOI:** 10.1155/2014/105428

**Published:** 2014-04-09

**Authors:** Sebastián A. Ríos, Ricardo Muñoz

**Affiliations:** Industrial Engineering Department, University of Chile, Av. República 701, 8370439 Santiago, Chile

## Abstract

Understanding the underlying community structure is an important challenge in social network analysis. Most state-of-the-art algorithms only consider structural properties to detect disjoint subcommunities and do not include the fact that people can belong to more than one community and also ignore the information contained in posts that users have made. To tackle this problem, we developed a novel methodology to detect overlapping subcommunities in online social networks and a method to analyze the content patterns for each subcommunities using topic models. This paper presents our main contribution, a hybrid algorithm which combines two different overlapping sub-community detection approaches: the first one considers the graph structure of the network (topology-based subcommunities detection approach) and the second one takes the textual information of the network nodes into consideration (topic-based subcommunities detection approach). Additionally we provide a method to analyze and compare the content generated. Tests on real-world virtual communities show that our algorithm outperforms other methods.

## 1. Introduction

The community finding problem [1] is key to understanding several different problems in social networks and other areas, for example, how these evolve through time [2], how information spreads in (online) human networks, and spam detection [3], among others.

Most community finding algorithms perform hard clustering on human networks based only on structural criteria, such as finding clique structures. Therefore, every member of a social network is assigned to only one community. However, we know that people can belong to more than one community (or subset of people with the same interests). Another shortcoming of these algorithms is that they ignore the content generated by the members of the community, where every post can let us improve the detection of the communities to which a member belongs. Methodologies showing that topics can be used to understand the dynamics of community structures are presented in Ding [4] and Yan et al. [5]. However, these methodologies are not comparable with our work. Thus, we decided to develop an algorithm to detect such communities allowing a member to participate in more than one community including semantic information. This is known as the overlapping community discovery problem. We benchmarked several algorithms, and we selected the best two algorithms for evaluating our algorithm, which are COPRA [6] and speaker-listener propagation algorithm (SLPA) [7]. We also used the speaker-listener topic propagation algorithm (SLTA), created by our group, which also performs well, as reported in [8].

Since 2009 we have stated the need for adding the semantic information of written comments by community members in order to perform online social network analysis (SNA) in a better (closer to reality) manner. This means extracting meaningful information leaving aside interactions which do not contribute to the main topics of human communication that are called online social network noise. We have proposed semantic filters to tackle this problem, reducing noise and showing good results in identifying key-members (central nodes) [9, 10], detecting preferences [11], and detecting a community (without overlapping) [12], among others.

This time, our main contribution is a new community finding algorithm (TPA) which allows members to belong to more than one community. Our algorithm (TPA) considers both structural properties of posted messages and the semantic information of posted content. We compared TPA to the best state-of-the-art algorithms, discovering that TPA outperforms them. In addition, we incorporate a method to interpret the content generated in each of the identified subcommunities, which is very useful when the activity in a social network is described.

This paper is structured as follows. In Section 2, previous work on detecting context-dependent communities in social networks is introduced. In Section 3, the proposed methodology and TPA algorithm are presented. In Sections 4 and 5 we present an experimental setup for an online community, and its results are discussed. Finally in Section 6, we establish the main conclusions of this work and indicate directions for future research.

## 2. Related Work

A great number of algorithms have been developed using a variety of methods [1, 13–15]; these vary in their effectiveness and time performance for different types of networks. This section summarizes three algorithms for overlapping community detection. Then, algorithms are benchmarked in unsupervised manner; therefore, we used link-based modularity [16] as a clustering quality measure for these algorithms.

### 2.1. Algorithms

#### 2.1.1. Community Overlap PRopagation Algorithm (COPRA)

COPRA (http://www.cs.bris.ac.uk/~steve/networks/software/copra.html) [6] is an algorithm based on the label propagation technique of Raghavan, Albert, and Kumara, but it is able to detect communities that overlap. Like the original algorithm, vertices have labels that propagate between neighboring vertices so that community members reach a consensus on their community membership, each node updating the coefficients that belong to it by averaging the coefficients from all its neighbors at each time step in a synchronous fashion.

#### 2.1.2. Speaker-Listener Propagation Algorithm (SLPA)

SLPA (https://sites.google.com/site/communitydetectionslpa/) [7] is a general speaker-listener based information propagation process. It spreads labels between nodes according to pairwise interaction rules. Unlike other algorithms, where a node forgets information gained in previous iterations, SLPA provides each node with a memory for storing received information (i.e., labels). The membership strength is interpreted as the probability of observing a label in a node's memory. In SLPA we need to determine the following: (1) how to spread node information to other nodes and (2) how to process the information received from other nodes. The critical issue related to both questions is how information should be maintained.

#### 2.1.3. Speaker-Listener Topic Propagation Algorithm (SLTA)

SLTA [8, 17] is a modification of the speaker-listener propagation algorithm (SLPA) [7]. In SLPA, the memory of each node is initialized with the node's id. SLTA follows this idea but applies a different initialization process. SLTA mimics human pairwise communication behavior. At each communication step, each node serves as both a speaker (information provider) and a listener (information consumer). Specifically, each node broadcasts a topic of interest to neighbors and at the same time receives an indication of interest from each neighbor.

### 2.2. Evaluation Criteria: Link-Based Modularity

To measure the quality of a cover (a cover of network is defined as a set of clusters such that each node is assigned to one or more clusters and no cluster is a proper subset of any other cluster) produced by overlapping detection algorithms on real-world social networks, where the ground truth is usually unknown, most measures extend the framework of modularity *Q* for a disjoint partition [18], which is given as

(1) $$\begin{array}{c} Q=\frac{1}{2m}\underset{c}{\sum }\underset{i,j\in c}{\sum }\left[A_{ij}-\frac{k_{i}k_{j}}{2m}\right], \end{array}$$

where *c* is a community, *A*_*ij*_ is the element of the adjacency matrix for nodes *i* and *j*,  *m* = (1/2)∑_*ij*_ *A*_*ij*_ is the total number of edges, and *k*_*i*_ is the degree of node *i* (each network node corresponds to an individual in the community).

In this paper we will use an extension of modularity based on the belonging coefficients of links proposed by Nicosia et al. [16]; this extension of modularity is used to evaluate the goodness of overlapped community decomposition. The Nicosia modularity is defined as

(2) $$\begin{array}{c} Q_{ov}^{Ni}=\frac{1}{m}\underset{c}{\sum }\underset{i,j\in V}{\sum }\left[\beta_{l(i,j),c}A_{ij}-\beta_{l(i,j),c}^{\text{out}}\beta_{l(i,j),c}^{\text{in}}\frac{k_{i}^{\text{out}}k_{j}^{\text{in}}}{m}\right], \end{array}$$

where *β*_*l*(*i*,*j*),*c*_ is the community belonging coefficient *c* of an edge *l* = (*i*, *j*) which starts at node *i* and ends at node *j*. *β*_*l*(*i*,*j*),*c*_^out^ is the expected belonging coefficient of any possible link *l*(*i*, *j*) from node *i* to node *j* in community *c* and *β*_*l*(*i*,*j*),*c*_^in^ is the expected belonging coefficient of any link *l*(*i*, *j*) pointing to node *j* in community *c*. *k*_*i*_^out^ and *k*_*i*_^in^ are the out-degree and the in-degree of node *i*, respectively, and *V* is the set of nodes. Note that if the modularity of an algorithm in a community equals zero, it implies that the algorithm did not find a community structure on this community and its output is a cluster containing all the community members.

## 3. Proposed Model

This section is organized as follows: first, basic notation and representation of documents are introduced; then, probabilistic models, network configuration, topic-based network filtering, and a modified community detection algorithm are presented.

### 3.1. Basic Notation and Concepts

Let us introduce some concepts. In the following, let *𝒱* be a vector of words that defines the vocabulary to be used. We will refer to a word *w*, as a basic unit of discrete data, indexed by {1,…, |*𝒱*|}. A posted message is a sequence of *S* words defined by **w** = (*w* ^1^,…, *w* ^*S*^), where *w* ^*s*^ represents the *s*th word in the message. Finally, a corpus is defined by a collection of *𝒫* posted messages denoted by *𝒞* = (**w**_1_, ..., **w**_|*𝒫*|_).

Social networks and communities have been studied by sociologists for many decades. They have proposed the following types of communities: communities of interest, communities of purpose, and communities of practice. In our previous work, we showed that our methodology enhances community detection in communities of practice (CoP). Thus, to determine whether these results can be replicated with other communities we focused on communities of interest (CoI) which have been studied by many researchers such as Kosonen [19] and Porter [20]. The dark web corresponds to virtual communities of interests (VCoI) [19, 20]. It gathers together groups of members whose interests are shared on different levels by the community users [10].

### 3.2. Topic Modeling

A topic model, for example, latent Dirichlet allocation (LDA) [21], can be considered to be a probabilistic model that relates documents and words through variables which represent the main topics inferred from the text itself. In this context, a document can be considered as a mixture of topics, represented by probability distributions which can generate the words in a document given these topics. The inferring process of the latent variables, or topics, is the key component of this model, whose main objective is to learn from text data the distribution of the underlying topics in a given corpus of text documents.

With LDA, given the smoothing parameters *β* and *α* and a joint distribution of a topic mixture *θ*, the idea is to determine the probability distribution to generate—from a set of topics *𝒯*—a message composed by a set of *S* words *w* (**w** = (*w* ^1^,…, *w* ^*S*^)),

(3) $$\begin{array}{c} p\left(\theta ,z,w\;|\;\alpha ,\beta \right)=p\left(\theta \;|\;\alpha \right)\overset{S}{\underset{s=1}{\prod }}p\left(z_{s}\;|\;\theta \right)p\left(w^{s}\;|\;z_{s},\beta \right), \end{array}$$

where *p*(*z*_*s*_ | *θ*) can be represented by the random variable *θ*_*i*_; this topic *z*_*s*_ is present in document *i* (*z*_*s*_^*i*^ = 1). A final expression can be deduced by integrating (3) over the random variable *θ* and summing topics *z* ∈ *𝒯*.

### 3.3. Network Configuration

To build the social network graph, the members' interaction must be taken into consideration. In general, member activity is followed according to participation in the forum. Likewise, participation appears when a member posts a comment in the community. The network will be configured according to the following:* nodes* will represent VCoI members and* arcs* will represent interactions among them. How to link the members and how to measure their interactions to complete the network is our main concern.

In this work we used an* all-previous-reply network* [9, 10, 22] to represent the VCoI network. This means when a member creates a post in a thread, every reply following it will be relayed to all the people who replied before on the thread. In other words, we assume that the last reply is a broadcast to all members who posted a comment before in that specific thread. This type of network representation is the densest and with the greatest number of interactions, and also the one with the most noisy arcs. It is therefore the hardest on which to apply data mining or social network analysis (SNA), and from which to extract useful information.

### 3.4. Topic-Based Network Filtering

The main idea of semantic filters is to compare semantic information of two members' posts with Euclidean distance. The semantic is extracted or represented by topics, which are not keywords. If the similarity is over a certain threshold *θ*, an interaction will be considered between them. We support the idea that this will help avoid irrelevant interactions. For example, in a VCoI with *k* topics, let TB_*j*_ be a post of user *j* that is a reply to post of user *i* (TB_*i*_). The cosine similarity between them will be calculated with

(4) $$\begin{array}{c} d_{m}\left({\text{TB}}_{i},{\text{TB}}_{j}\right)=\frac{\sum_{k}g_{ik}g_{jk}}{\sqrt{\sum_{k}g_{ik}^{2}\sum_{k}g_{jk}^{2}}}, \end{array}$$

where *g*_*ik*_ is the score of topic *k* in the post of user *i* (the topic-post distribution obtained with LDA). It is clear that the similarity exists only if TB_*j*_ is a reply to TB_*i*_. After that, the weight of arc *a*_*i*,*j*_ is calculated according to

(5) $$\begin{array}{c} a_{i,j}=\underset{\begin{array}{c} i,j\\ d_{m}\left(\text{T}{\text{B}}_{i},\text{T}{\text{B}}_{j}\right)\geq \theta \end{array}}{\sum }d\left(\text{T}{\text{B}}_{i},\text{T}{\text{B}}_{j}\right). \end{array}$$

Considering all posts *𝒫*, the network is built following the structure described in Section 3.3. In other words, for each post of user *i* in a thread, the arc *a*_*i*,*j*_ is added for each user *j* who posted a comment on that thread. But we only consider the arcs if the similarity of their messages is greater than or equal to the threshold *θ* in (5). This way, we are able to filter arcs by topic similarity to a specific thread's topics.

### 3.5. Community Detection in Topic-Based Networks, Topic Propagation Algorithm (TPA)

This algorithm extends the idea presented by Ríos and Muñoz [8] where the topic used most for a node is propagated in the network. In this algorithm, nodes interact among themselves following a certain interaction rule, which updates the membership vector of each node in an asynchronous process. The membership vector for each node is initialized with its topic score's vector (see (6)). In summary, the proposed algorithm consists of the following three stages.

1. The membership vector of each node is initialized with its topic score's vector. The topic score's vector is computed using text mining techniques, specifically LDA, which was applied in the topic modeling step (see Section 3.2).
2. Then, the following steps are repeated until the stop criterion is satisfied.
  - One node is selected as a candidate.
  - The average membership vector of all neighbors of the selected node is calculated.
  - The candidate updates its membership vector following a certain interaction rule between its membership vector and the average membership vector of its neighbors. Then, the candidate's membership vector is normalized.
3. Finally, the postprocessing based on the belonging vectors of nodes is applied to output the communities.

In the initialization process, the membership vector of a node *i* is initialized with its topic score's vector Ψ^*i*^; Ψ^*i*^ is a vector where every component is the average score over all post messages from user *i*. Mathematically,

(6) $$\begin{array}{c} \Psi_{k}^{i}=\frac{1}{\left|\text{TB}\right|}\underset{l\in \text{TB}}{\sum }q_{ikl} \end{array}$$

(7) $$\begin{array}{c} \Psi^{i}=\frac{1}{||\Psi^{i}||}\Psi^{i}, \end{array}$$

where TB is the set of users' posted messages; *q*_*ikl*_ is the score of topic *k* in post *l* of user *i*.

Let Ψ_*n*_^*i*^ be the normalized average membership vector of node *i*'s neighborhood. Then, the interaction rule between Ψ^*i*^ and Ψ_*n*_^*i*^ at iteration *t* updates the belonging vector of node *i* as follows:

(8) $$\begin{array}{c} \Psi^{i,t}=\Psi^{i,t-1}+\varphi \left(\Psi^{i,t-1},\Psi_{n}^{i,t-1}\right)\left[\Psi_{n}^{i,t-1}-\Psi^{i,t-1}\right]\\ \Psi^{i,t}=\frac{1}{||\Psi^{i,t}||}\Psi^{i,t}. \end{array}$$

After a modularity optimization process, we estimated the function *φ*(Ψ^*i*^, Ψ_*n*_^*i*^) as

(9) $$\begin{array}{c} \varphi \left(\Psi^{i},\Psi_{n}^{i}\right)=\exp\left(-\frac{t}{4\;\log\left(k\right)}\right). \end{array}$$

(*a) Stopping Criterion*. We can stop at any time as long as we collect sufficient information for postprocessing. In the current implementation we simply stop when the predefined maximum number of iterations *T* is reached. Although TPA is nondeterministic due to the random selection, it performs well on average as shown in later sections. This algorithm produces relatively stable outputs, independent of network size or structure, when *T* is greater than 20.

(*b) Postprocessing and Community Detection.* Given the membership vector of a node/member, a simple thresholding procedure is performed to produce an overlapped assignment of members to different communities. If the topic score of certain component is greater than a given threshold *r* ∈ [0,1], this node belongs to those communities. Thus, that node is called an overlapping node.

### 3.6. Community Characterization

The aim of community characterization is to find out what the community is talking about. For this purpose, we propose the following methodology. Let *T* = VC(*V*, *E*) ∈ TVC be a virtual community and *C*_1_, *C*_2_,…, *C*_|*C*|_ a set of cover for virtual community *T*. For each cover *C*_*j*_ we define the content community pattern *CV*_*j*_, a vector of length *K*, where *K* is number of topics extracted for corpus *𝒞*. The component *CV*_*j*_^*k*^ can be expressed as follows:

(10) $$\begin{array}{c} CV_{j}^{k}=\frac{\left|cv_{j}^{k}\right|}{\sum_{j\in C}\left|cv_{j}^{k}\right|}, \end{array}$$

where *cv*_*j*_^*k*^ = {*P*_*il*_ ∈ TB | *q*_*ikl*_ ≥ *κ*  and  *i* ∈ *C*_*j*_} and *cv*_*j*_^*k*^ is the set of posts written by members of community *j* who has a topic score *q*_*ikl*_ for topic *k* greater than a threshold *κ* ∈ [0,1].

## 4. Experimental Setup and Results

The method presented in Section 3.5 was evaluated over a dark web portal dataset. The dark web forum portal [23] is a web-based knowledge portal which was created based on a general framework for web forum data integration. The portal incorporates the data collected from different international Jihadist forums. These online discussion sites are dedicated to topics relating primarily to Islamic ideology and theology. The dark web can be considered to be a virtual community of interests (VCoI) whose members are extremists who share and comment on their feelings and interests with others who support their cause. Our proposed methodology for overlapping community detection was applied to the  IslamicAwakening English language based forum, available on ISI-KDD 2012 Website (http://www.ischool.drexel.edu/isi-kdd2012/challenge.html).

Next, an analysis of topics extracted using LDA (described in Section 3.2) is presented. Then, the network topology construction is described by using* all-previous-reply* oriented structures for the whole period. Finally, overlapping community detection algorithms were applied, and their results were compared with different LDA-filtered networks.

In order to validate the proposed method (described in Section 3.5), we applied this method using five different LDA-filtered networks. To better understand the performance of the proposed algorithm, we compared TPA with two well-known algorithms, COPRA [6] and SLPA [7]. We also compared TPA with SLTA [8, 17], a modified version of SLPA which includes semantic information.

We used default parameter settings for most algorithms where applicable. For TPA the maximum number of iterations *T* was set to 30, and for SLPA and SLTA, this parameter was set to 100. The threshold *r* for SLPA and SLTA takes values in set {0.01,0.05,0.1,0.2,0.3,0.4} and for TPA, parameter *r* varies from 1/*k* to 5/*k* with an interval 1/2 *k*, where *k* is the number of extracted topics. For the extracted communities we measured and reported the maximum performance over ten repetitions for TPA, SLPA, SLTA, and COPRA.

We selected overlapping link-based modularity *Q*_*ov*_^*Ni*^ in (2) as a quality measure. Although modularity is a function of a cover and a network, we sometimes refer to the* modularity of algorithm A on network N*, referring to the modularity of the cover produced by algorithm A when run on network N.

### 4.1. Topic Extraction

There are 7 years (2004–2010) of data available. Posts were created by 2,792 members and extracted topics were realized over 127,216 posts *𝒫* and 244, 200 words in the vocabulary *𝒱* by using a Java Gibbs sampling-based implementation of LDA (http://jgibblda.sourceforge.net/) previously described in Section 3.2.

The application of LDA over text content resulted in {10,50,100} topics with 20 words and their respective probabilities. The most popular topics extracted from the  IslamicAwakening forum are presented in Table 1. These topics represent the most popular ideas posted in the forum when 100 topics are extracted.

### 4.2. Topic-Based Social Network Visualization

The graph has many variables which modify its configuration [22].

1. *Time.* One dimension that was not mentioned before is time. The time period to be analyzed could be a month, annually, or whole history of posts of the network.
2. *Graph Filtering.* Including the traditional nonfiltered graph, the other four configurations correspond to graphs filtered by topics, as presented in Section 3.4.
3. *Interaction Topology.* According to the assumption of* who* is replying, the all-previous-reply network is considered. In this configuration every reply of a thread will be a response to all posts which are already in a specific thread.

To configure the network and have the graph representation, all of these three variables have to be decided. In this work, three temporal virtual communities (TVC) of interest are used. They are extracted from the same forum data, but they have different time frames. We used 20 four-month time frames for the period between January 2004 and August 2010.

Networks were built from 2004 to 2010 using three different topic sets, and for comparisons, the threshold *θ* takes values in set {0.0, 0.1, 0.2, 0.3, 0.4} for LDA-Filter, as explained in Section 3.4. Then, for each interaction representation, the result is a graph with the members who posted in a specific period of time and has an interaction greater than or equal to the filter threshold. We chose the LDA threshold to eliminate a large number of irrelevant interactions but without excluding many members from the network. For example, after applying the highest threshold over the one-year TVC, the networks have, on average, 25% fewer interactions but we excluded only 5% of the members.

## 5. Overlapping Community Detection

**Figure 1:**
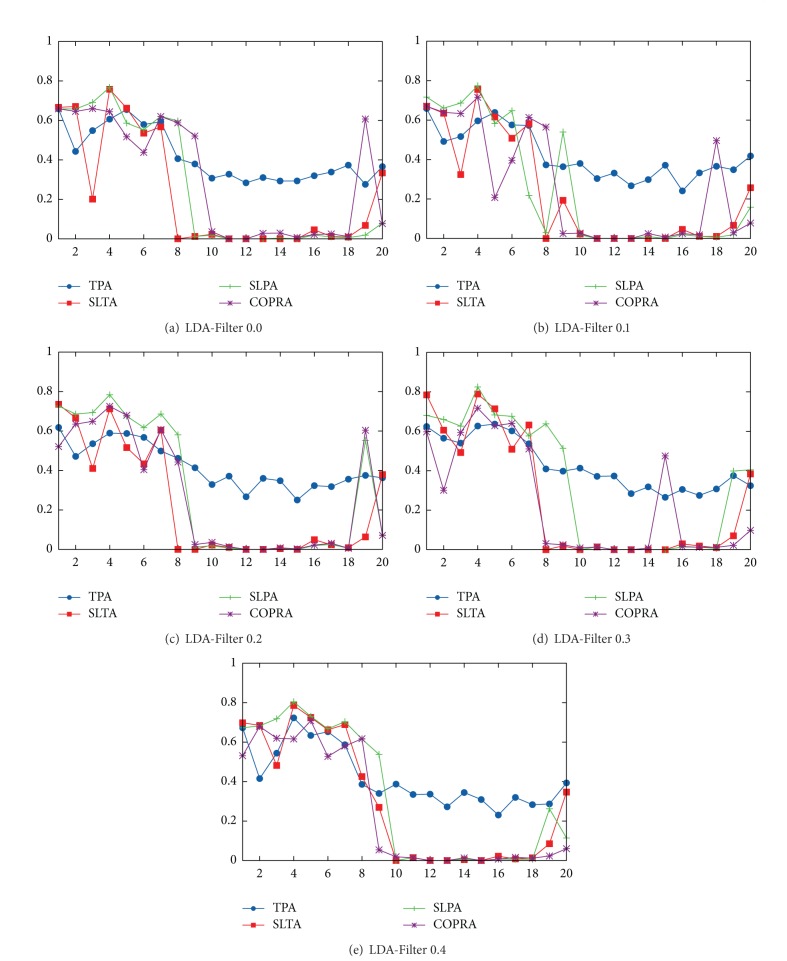
Algorithm modularity comparison for four-month TVC. (Figures include the period from Jan. 2004 to Aug. 2010.)

Our results are shown in Figure 1. Figure 1 shows results obtained after applying the methodology described in Section 3 for all four-month TVCs available. This figure shows the maximum link-based modularity (*Q*_*ov*_^*Ni*^, see Section 2.2) over ten repetitions for algorithms considered in this work. Despite using three different topic sets to detect overlapping communities, we only present those results with the highest average modularity which were obtained using 50 topics.

Results show that TPA achieves, on average, a modularity measure of 0.33 while the best state-of-the-art algorithm achieves only 0.043 when it is applied over a VCoI. Nonetheless, there are no different results when the semantic filter applied over the networks is increased. We excluded results with a modularity measure that equals zero from this analysis because of the distortion that occurs when the average is calculated.

According to sociological theory, it is expected that VCoI's members are related to one another because they share the same interests. Therefore, all users should belong to the same community. Most algorithms capture this; they find only a cover which contains all members. Nonetheless, TPA is able to detect several subcommunity structures. We will see below that detected community structures define groups of members that share almost all the content generated within them, and they differ only in a few topics while most state-of-the-art algorithms cannot detect these groups because they do not include semantic information as TPA.

In Figure 1 we do not observe a clear advantage of one algorithm over the other in the period between 2004 and 2006 (see abscissa points from 1 to 9). However, TPA achieves a clear advantage for the period between 2007 and 2010 (see abscissa points from 10 to 20), because during this period the VCs become denser, making it more difficult for traditional algorithms to find a community structure using only structural properties, and discovered subcommunities are only explained due to subtle changes in the content spoken in each one of them, which is captured by TPA. We obtained similar TPA behavior regardless of the semantic filter (*θ*) applied. Remember that if the modularity of some algorithm on a VC equals zero, it implies that the algorithm did not find a community structure on this VC and its output is a cluster containing all the VC's nodes. This means that our algorithm systematically discovers overlapping communities, while other algorithms do not discover any community structures.

**Figure 2:**
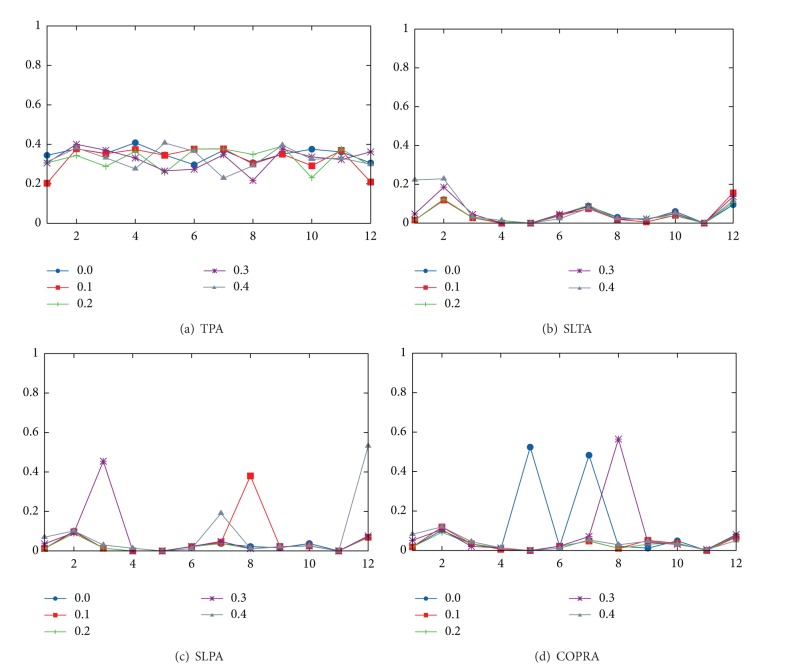
Effects of different semantic LDA-Filter on algorithms using one-month TVC (we include 2009).

After analyzing the effect of different semantic filters on the quality of overlapping community detection on VCoI, it is possible to see that there are no significant differences. In Figure 2 we illustrate the effect of LDA-Filters on overlapped community detection performance using one-month TVC for the year 2009.

*Findings.* The former is very important since in a previous work using virtual communities of practice (VCoP) [17] we discovered that semantic filters do affect overlapping community detection performance. In other words, the modularity of SLTA and SLPA is higher when the semantic threshold is higher; that is, irrelevant interactions are deleted. Therefore, less noise is processed and more communities are found. This means that the type of virtual community being processed affects the outcome of the algorithms.

**Figure 3:**
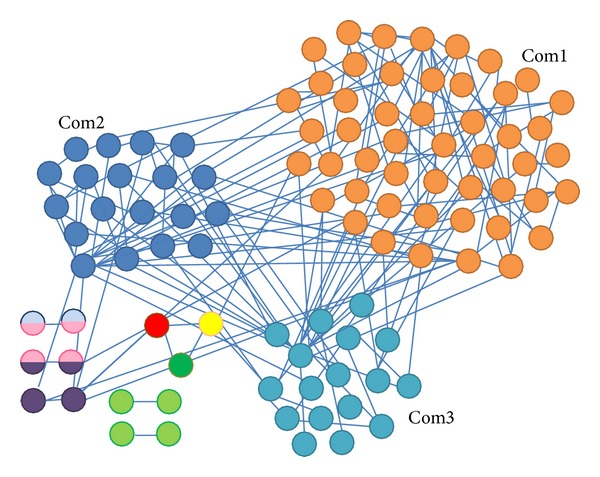
Dark web portal 2005 LDA-filtered network, with LDA-Filter 0.1.

We can state that we have changed the actual idea of community, requiring that at least two nodes have an edge connecting each other to belong to the same community. In our case, we extended that notion since—in addition to a connected subgraph—we also used the members' interaction semantic information, which means that two nodes belong to the same community if they are also interested in the same topics. This allows us to identify better groups and find much better communities, where every community is characterized according to the methodology developed in this work (see Section 3). In Figure 3, we show communities detected through TPA for a 2005 data network (LDA-Filter 0.1). Multicolor nodes indicate overlap nodes/members. This figure illustrates the new concept of community where two nodes do not need to be connected to belong to the same community, while they share the same interest. Our object is that this new definition of community will allow obtaining results that are closer to reality while gathering better information for analysts. This new information can be used from recommender systems to community management or even community moderation.

### 5.1. Community Characterization

In this section, detected overlapping communities are characterized according to the methodology proposed in Section 3.6.

**Figure 4:**
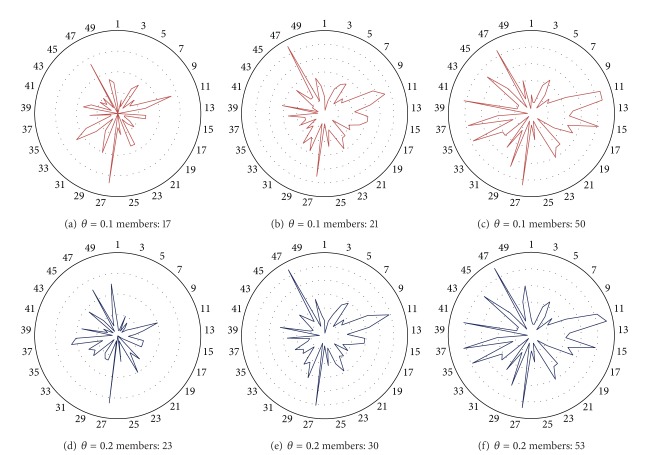
Content community pattern of the three largest communities detected through TPA on 2005-VC.

Figure 4 shows the content pattern for the three largest communities detected in filtered 2005-VC with LDA-Filter *θ* = 0.1 and *θ* = 0.2. Each content pattern shows the topic distribution over each community. In this case, it is possible to see how different content patterns are when communities for the same filtered VC are analyzed; this is likely to happen because the communities were formed towards different interest topics. On the contrary, when communities of similar size are analyzed across different filtered 2005-VC it is possible to see that the content for each does not change when we apply different semantic filters. These properties are expected of any semantic-based community description. First, it is expected that detected communities in the same VC are described by different topic distributions. Finally, the content, that is, topic distribution within a community, is independent of irrelevant interactions. Therefore, topic distribution for a community does not change when a semantic filter is applied, since its topics are always its topics.

**Figure 5:**
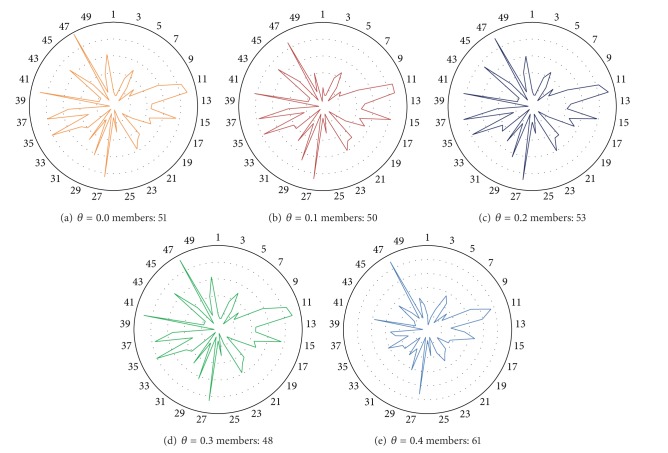
Content community patterns of the largest communities detected through TPA on 2005-VC with several semantic filters.

After applying TPA algorithm for each LDA-filtered network, the content community pattern for the largest community detected remains the same (see Figure 5). Despite the fact we applied different semantic filters, it is possible to see that there is no change in community content pattern. This example corroborates the idea explained above; the community content pattern is constant even if a semantic filter is applied. This can be explained for the methodology used to characterize a community, where we only included components over a threshold in our analysis. This lets us avoid the inclusion of irrelevant topics within a community, leaving only the core meaning of every post.

As an example we applied the methodology described in Section 3.6 to 2005-VC with a semantic filter *θ* = 0.1. We characterized the TPA's output which obtained the highest modularity after ten iterations.

Table 2 shows the three largest communities detected based on the 2005-VC with LDA-Filter 0.1 by the TPA community detection approach. For each community, the five topics with the highest score are shown. In this example, we can see that, within the five most relevant topics for each community, three of them are shared by all communities ((1)* live life quotes, *(2)* profiles (forum), and *(3)* Islam in general*) and just two of them let us characterize the content generated by community members.

## 6. Conclusions

Community discovery on social networks is a hot topic which affects many different areas from recommender systems to community management or even community moderation. However, most algorithms detect hard communities, which means that every member belongs to only one discovered community. This is far from reality since most people have more than one interest; therefore, they usually belong to more than one community. This is called the overlapping community detection problem and only a few algorithms exist.

In this paper, we report our work in which we developed a new overlapping community discovery algorithm called the* topic propagation algorithm* (TPA) to tackle the problem of overlapping community detection. Our algorithm incorporates the sociological point of view that people have multiple interests and that communities reflect those interests. Thus, our algorithm takes advantage of a topic model, semantic filters, and social network representation.

We benchmarked our algorithm with COPRA, SLPA, and SLTA, which are state-of-the-art overlapping community detection algorithms. We tested all algorithms using a real-world dataset corresponding to a virtual community of interest (VCoI).

Experiments show that modularity results for TPA discovering overlapping communities outperform those of SLPA, SLTA, and COPRA. We also systematically obtained better results when modifying the time frame analyzed or the semantic filter threshold.

When our methodology is applied in order to detect overlapping communities over a VCoI, the expectation is that it will not detect subcommunity structure because all VCoI's members only share ideas and thoughts on a common interest or passion but they do not share their knowledge and expertise to learn more about a specific topic. After applying our methodology we validated the underlying theory, finding that our algorithms do not detect a better subcommunity structure when the semantic filter is increased over a VCoI. Moreover, only TPA is capable of finding a subcommunity structure on a VCoI because TPA includes semantic information to detect overlapping communities and it is able to capture groups which differ only in a few topics.

## Figures and Tables

**Table 1:** Ten most relevant words with their respective conditional probabilities for the five most relevant topics for all data from the I slamicAwakening Forum.

Topic 19	Topic 15	Topic 33	Topic 54	Topic 51
“War”	“Terrorist attacks (general)”	“Political/terrorist trials”	“Religion (Allah)”	“Islamic religion”
Government (0.0140)	Kill (0.0255)	Court (0.0149)	Allah (0.0141)	Islamic (0.0254)
War (0.0113)	Police (0.0180)	Guantanamo (0.0099)	Prophet (0.0163)	Muslim (0.0176)
Military (0.0104)	Soldier (0.0168)	Trail (0.0080)	Messenger (0.0141)	Islam (0.0159)
Country (0.0091)	Attack (0.0139)	Prison (0.0073)	Peace (0.0118)	World (0.0126)
United (0.0089)	Force (0.0137)	Judge (0.0066)	People (0.0113)	Religious (0.0107)
Security (0.0083)	Military (0.0109)	Torture (0.0065)	Lord (0.0107)	Society (0.0104)
Force (0.0079)	Official (0.0105)	Rights (0.0061)	Day (0.0099)	People (0.0086)
International (0.0059)	Security (0.0089)	Charges (0.0057)	Believer (0.0096)	Law (0.0083)
Official (0.0059)	Report (0.0086)	Government (0.0056)	Bless (0.0084)	Political (0.0078)
American (0.0056)	Army (0.0075)	Arrested (0.0056)	Quran (0.0077)	Western (0.0070)

**Table 2:** Three largest communities for 2005-VC (0.1).

Community	Topic	Top 10 words
Largest community members = 50	Topic 12 (0.043)	Ibn, hadith, imam, abu, book, narration, ahmad, al, weak, Muhammad
	Topic 11 (0.043)	People, time, feel, talk, person, bad, life, understand, live, start
	Topic 27 (0.043)	Allah, people, lord, heart, love, believer, day, person, life, prophet
	Topic 47 (0.043)	Quote, posted, originally, bro, akhi, abumuwahid, ahmed, brothermujahid, waziri, wild
	Topic 40 (0.041)	Sheikh, al, ibn, Muhammad, scholar, bin, Abdullah, book, lecture, anwar

Second community members = 21	Topic 47 (0.055)	Quote, posted, originally, bro, akhi, abumuwahid, ahmed, brothermujahid, Waziri, wild
	Topic 11 (0.045)	People, time, feel, talk, person, bad, life, understand, live, start
	Topic 27 (0.045)	Allah, people, lord, heart, love, believer, day, person, life, prophet
	Topic 10 (0.038)	Salafi, people, call, sunnah, dawah, issue, aqeedah, scholar, qutb, manhaj
	Topic 12 (0.033)	Ibn, hadith, imam, abu, book, narration, ahmad, al, weak, Muhammad

Third community members = 17	Topic 27 (0.067)	Allah, people, lord, heart, love, believer, day, person, life, prophet
	Topic 47 (0.054)	Quote, posted, originally, bro, akhi, Abumuwahid, ahmed, brothermujahid, waziri, wild
	Topic 11 (0.054)	People, time, feel, talk, person, bad, life, understand, live, start
	Topic 34 (0.047)	Sufi, music, sheikh, tasawwuf, love, listen, people, sound, call, singing
	Topic 29 (0.040)	Alaykum, assalam, wa, salam, inshallah, assalamu, rahmatullah, hope, forum, false

## References

[1] Mitchell M (2006). Complex systems: network thinking. *Artificial Intelligence*.

[2] Prieto B, Tricas F, Merelo JJ, Mora A, Prieto A (2008). Visualizing the evolution of a web-based social network. *Journal of Network and Computer Applications*.

[3] Sadan Z, Schwartz DG (2011). Social network analysis of web links to eliminate false positives in collaborative anti-spam systems. *Journal of Network and Computer Applications*.

[4] Ding Y (2011). Community detection: topological vs. topical. *Journal of Informetrics*.

[5] Yan E, Ding Y, Milojević S, Sugimoto CR (2012). Topics in dynamic research communities: an exploratory study for the field of information retrieval. *Journal of Informetrics*.

[6] Gregory S (2010). Finding overlapping communities in networks by label propagation. *New Journal of Physics*.

[7] Xie J, Szymanski BK, Liu X, Spiliopoulou M, Wang H, Cook DJ SLPA: uncovering overlapping communities in social networks via a speaker-listener interaction dynamic process.

[8] Ríos SA, Muñoz R Dark web portal overlapping community detection based on topic models.

[9] Alvarez H, Ríos SA, Aguilera F, Merlo E, Guerrero L (2010). Enhancing social network analysis with a conceptbased text mining approach to discover key members on a virtual community of practice. *Knowledge-Based and Intelligent Information and Engineering Systems*.

[10] L’Huillier G, Alvarez H, Aguilera F, Ríos SA Topic-based social network analysis for virtual communities of interests in the dark web.

[11] Ríos SA, Aguilera F, Guerrero L (2009). Virtual communities of practice’s purpose evolution analysis using a concept-based mining approach. *Knowledge-Based Intelligent Information and Engineering Systems-Part II*.

[12] Cuadra L, Ríos SA, L'Huillier G, Hübner JF, Petit JM, Suzuki E (2011). Enhancing community discovery and characterization in vcop using topic models. *Web Intelligence/IAT Workshops*.

[13] Li D, Ding Y, Shuai X (2012). Adding community and dynamic to topic models. *Journal of Informetrics*.

[14] Shen H, Cheng X, Cai K, Hu M (2009). Detect overlapping and hierarchical community structure in networks. *Physica A: Statistical Mechanics and its Applications*.

[15] Wu Z, Lin Y, Wan H, Tian S, Hu K (2012). Efficient overlapping community detection in huge real-world networks. *Physica A: Statistical Mechanics and its Applications*.

[16] Nicosia V, Mangioni G, Carchiolo V, Malgeri M (2009). Extending the definition of modularity to directed graphs with overlapping communities. *Journal of Statistical Mechanics: Theory and Experiment*.

[17] Muñoz R, Ríos SA, Grana M, Toro C, Posada J, Howlett RJ, Jain LC (2012). Overlapping community detection in vcop using topic models. *KES*.

[18] Newman MEJ (2004). Fast algorithm for detecting community structure in networks. *Physical Review E—Statistical, Nonlinear, and Soft Matter Physics*.

[19] Kosonen M (2009). Knowledge sharing in virtual communities—a review of the empirical research. *International Journal of Web Based Communities*.

[20] Porter CE (2004). A typology of virtual communities: a multi-disciplinary foundation for future research. *Journal of Computer-Mediated Communication*.

[21] Blei DM, Ng AY, Jordan MI (2003). Latent dirichlet allocation. *Journal of Machine Learning Research*.

[22] Alvarez H (2010). *Deteccion de miembros clave en una comunidad virtual de pratica mediante analisis de redes sociales y minerıa de datos avanzada (key-members detection in a virtual community of practice using social network analysis and advanced data mining) [M.S. thesis]*.

[23] Zhang Y, Zeng S, Fan L, Dang Y, Larson CA, Chen H Dark web forums portal: searching and analyzing jihadist forums.

