# Corrigendum to “Content Patterns in Topic-Based Overlapping Communities”

**DOI:** 10.1155/2016/4162801

**Published:** 2016-08-23

**Authors:** Sebastián A. Ríos, Ricardo Muñoz

**Affiliations:** Industrial Engineering Department, University of Chile, Av. República 701, 8370439 Santiago, Chile

In the article titled “Content Patterns in Topic-Based Overlapping Communities”, [1] the “Acknowledgments” section was missing, which should be added as follows.
